# Algorithms for Automated Calibration of Transcutaneous Spinal Cord Stimulation to Facilitate Clinical Applications

**DOI:** 10.3390/jcm10225464

**Published:** 2021-11-22

**Authors:** Christina Salchow-Hömmen, Thomas Schauer, Philipp Müller, Andrea A. Kühn, Ursula S. Hofstoetter, Nikolaus Wenger

**Affiliations:** 1 Department of Neurology, Charité–Universitätsmedizin Berlin, 10117 Berlin, Germany; christina.salchow@charite.de (C.S.-H.); andrea.kuehn@charite.de (A.A.K.); nikolaus.wenger@charite.de (N.W.); 2 Control Systems Group, Technische Universität Berlin, 10587 Berlin, Germany; mueller@control.tu-berlin.de; 3 Center for Medical Physics and Biomedical Engineering, Medical University of Vienna, 1090 Vienna, Austria; ursula.hofstoetter@meduniwien.ac.at

**Keywords:** automation, electromyography, noninvasive, Parkinson’s disease, posterior root-muscle reflexes, spasticity, spinal cord injury, spinal cord stimulation, transcutaneous

## Abstract

Transcutaneous spinal cord stimulation (tSCS) is a promising intervention that can benefit spasticity control and augment voluntary movement in spinal cord injury (SCI) and multiple sclerosis. Current applications require expert knowledge and rely on the thorough visual analysis of electromyographic (EMG) responses from lower-limb muscles to optimize attainable treatment effects. Here, we devised an automated tSCS setup by combining an electrode array placed over low-thoracic to mid-lumbar vertebrae, synchronized EMG recordings, and a self-operating stimulation protocol to systematically test various stimulation sites and amplitudes. A built-in calibration procedure classifies the evoked responses as reflexes or direct motor responses and identifies stimulation thresholds as recommendations for tSCS therapy. We tested our setup in 15 individuals (five neurologically intact, five SCI, and five Parkinson’s disease) and validated the results against blinded ratings from two clinical experts. Congruent results were obtained in 13 cases for electrode positions and in eight for tSCS amplitudes, with deviations of a maximum of one position and 5 to 10 mA in amplitude in the remaining cases. Despite these minor deviations, the calibration found clinically suitable tSCS settings in 13 individuals. In the remaining two cases, the automatic setup and both experts agreed that no reflex responses could be detected. The presented technological developments may facilitate the dissemination of tSCS into non-academic environments and broaden its use for diagnostic and therapeutic purposes.

## 1. Introduction

Epidural electrical stimulation (EES) of the lumbar spinal cord has recently experienced a surge of interest because of its potential to restore voluntary control of locomotion in individuals after severe spinal cord injury (SCI) [1,2,3]. Through the recruitment of large-to-medium diameter proprioceptive and cutaneous afferents within lumbar and upper sacral posterior roots [4,5], EES can facilitate the alleviation of severe lower limb spasticity [6] and the generation or augmentation of rhythmic and locomotor-like lower limb activity in otherwise paralyzed legs in individuals with SCI [1,4,7,8]. Moreover, in other neurological disorders, treatment effects of EES are under active investigation. For example, in advanced Parkinson’s disease (PD), EES was shown to ameliorate motor symptoms such as impaired gait function and postural stability [9,10,11,12], yet a recent study could not reproduce these outcomes [13]. A general problem of EES is the lack of clinical or physiological markers for identifying treatment responders in advance [14].

**Figure 1:**
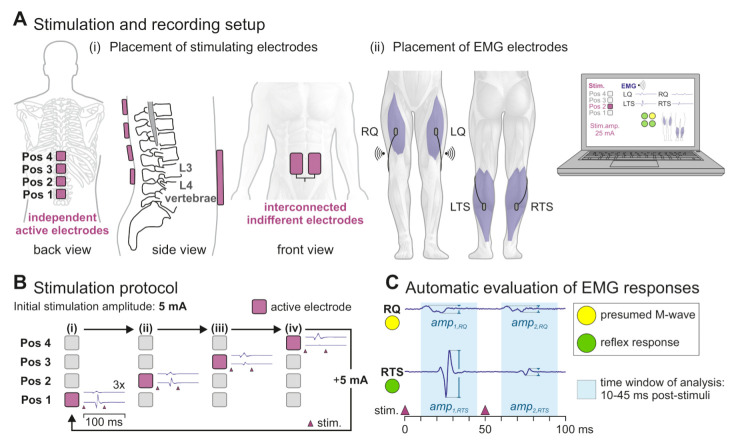
Automated stimulation and recording setup for lumbosacral posterior root stimulation. (**A**) (i) Separate active electrodes were placed at four rostrocaudal positions over the spine (Pos 1–4), with the most caudal electrode located between the L3 and L4 spinous processes. Two interconnected electrodes on the lower abdomen acted as common indifferent electrode. (ii) Surface electromyographic (EMG) recordings were acquired from right (R) and left (L) quadriceps (Q) and triceps surae (TS) muscle groups by using a two-channel sensor per lower limb. Information on the selected active electrode and the recorded EMG activity was transmitted to a laptop and displayed in a custom user interface. (**B**) The automated stimulation protocol systematically tested electrode positions 1–4 using three double stimuli per stimulation amplitude, starting with 5 mA. In each iteration of the protocol, the stimulation amplitude was increased by 5 mA up to a maximum of 75 mA or the individually maximally tolerated amplitude. (**C**) The custom user interface allowed for the visualization of averaged EMG waveforms of muscle responses elicited by the three double stimuli with a given amplitude, here exemplarily shown for RQ and RTS. Peak-to-peak amplitudes of the responses to the respective first and second stimuli were automatically computed ($amp_{1,\mathrm{RQ}}$, $amp_{2,\mathrm{RQ}}$, and $amp_{1,\mathrm{RTS}}$ and $amp_{2,\mathrm{RTS}}$). By applying the rules of the defined rating light system (Table 2), the responses were automatically classified as being a presumed M-wave or reflex response.

The target neural structures of lumbar EES [4,5] can also be recruited noninvasively by using transcutaneous spinal cord stimulation (tSCS) [15,16,17]. Transcutaneous SCS uses surface electrodes placed on the paravertebral and abdominal skin to generate a current flow through the lower trunk, partially crossing the dural sac [5,17] (see Figure 1). Independent studies have shown the efficacy of tSCS to ameliorate spasticity and augment voluntary motor control, including locomotion in individuals with SCI [18,19,20,21,22,23,24] as well as multiple sclerosis [25,26]. As a clinically accessible and noninvasive approach, tSCS was suggested to hold the potential to develop into a widely used neurorehabilitation technique and to serve as a screening tool to estimate individually attainable therapeutic outcomes of EES [23].

The application of tSCS has so far been restricted to specialized research centers, in part owing to the required expert knowledge and constraints in clinical time management. For example, a necessary prerequisite to induce neuromodulatory effects in the lower limbs by EES or tSCS is the specific placement of the epidural or surface electrodes, respectively, so as to overlie the spinal cord segments innervating lower extremity muscles [6,8,17,20,23,27]. Such placement can be validated via the electromyographic (EMG) recording of evoked responses, i.e., short-latency reflexes initiated within the posterior roots, so-called posterior root-muscle (PRM) reflexes [1,7,8,16,18,22,28,29,30,31]. These reflexes are generally thought to result from the activation of proprioceptive fibers within the posterior roots that cause synchronized responses of motoneurons in the spinal cord [32,33]. With respect to tSCS specifically, the placement of the paravertebral stimulating electrode typically follows a multi-step procedure. First, after rough palpation of the spinal column, a self-adhesive surface electrode is placed over the spine at a level estimated to correspond to the T11/T12 spinous processes [29]. Single stimulation pulses are then applied at incremental amplitudes with the aim to elicit PRM reflexes in L2–S2 innervated lower limb muscles [16,29]. In order to achieve this aim, the electrode may have to be relocated by several centimeters in either the rostral or caudal direction, and with each new position, the procedure of single stimulation pulse application is repeated. With the electrode placement eventually designated for therapy, double stimuli are applied, normally with interstimulus intervals of 30–100 ms, to test for the presence of post-stimulation attenuation of the evoked responses and, hence, identify them as reflexes [18,29]. In contrast, post-stimulation attenuation is not observed for EMG responses that result from the direct stimulation of efferent motor pathways (M-waves) in the anterior roots or peripheral nerves [18,29]. Finally, reflex response thresholds are determined based on available EMG recordings. In tSCS applications for spasticity control, the stimulation amplitude is then set to approximately 90% of the lowest PRM reflex threshold [18,23,25]. This time-consuming procedure likely imposes an impediment to the wider use of tSCS in real-world clinical practice. Furthermore, stimulation devices normally used in neurorehabilitation do not allow for the application of single and double stimuli, let alone for the synchronous recording of EMG activity from several muscles.

The aim of the present study was to cast this expert knowledge into an automated tSCS setup that identifies appropriate stimulation sites over the lumbosacral spinal cord and determines clinically suitable stimulation amplitudes for the application of tSCS in spasticity control [18,20,23,25]. To this end, we combined an electrode-array configuration, a self-operating stimulation protocol, synchronous recordings of EMG responses from several lower-limb muscles, and online evaluation algorithms. We tested our approach in neurologically intact individuals as well as in individuals with SCI and PD. The accuracies of the automated results were validated by two independent experts. To ease clinical decision making, we propose a graphical user interface that indicates optimal stimulation configurations with an intuitive color code. Our results may aid the dissemination of tSCS technologies into non-academic environments and broaden the use of tSCS for both diagnostic and therapeutic applications in the near future.

## 2. Materials and Methods

### 2.1. Participants

The automated tSCS setup was tested in five neurologically intact volunteers (mean age ± SD, 32.8 ± 2.77 years), five individuals with chronic SCI (47.0 ± 11.51 years), and five individuals with idiopathic PD (70.2 ± 6.42 years; Table 1). Among the exclusion criteria were any active implants or passive implants at vertebral level T8 or caudally. All procedures were approved by the Ethics Committee of the Berlin Chamber of Physicians (ETH-28/17; 2017 and 2019; SCI) and the Ethics Committee of the Charité–Universitätsmedizin Berlin (EA2/118/18, 2018; PD and healthy controls). All participants signed written informed consent forms prior to their enrollment into the study.

### 2.2. Stimulation and Recording Setup

Four separate hydrogel surface electrodes (each 5 × 5 cm; axion, Leonberg, Germany) were placed over the spine in a rostrocaudal arrangement [35], starting with the most caudal electrode that was positioned between the L3/L4 spinous processes (position 1) based on palpation (Figure 1A(i)). The remaining three electrodes were then placed rostrally along the spine (positions 2–4), with an inter-electrode distance of 1 cm. Such setup ensured coverage of the targeted lumbosacral posterior roots by at least one of the electrodes. In individuals C5, S5, and P5, only three electrodes were used due to their comparatively smaller body heights. Two interconnected hydrogel electrodes (each 7 × 12 cm; axion, Leonberg, Germany) were placed on the lower abdomen symmetrically to the umbilicus [17,18]. Each of the paraspinal electrodes could be separately selected as stimulating electrode, with the abdominal electrodes acting as the common indifferent electrode. Stimulation was applied via a four-channel electrical stimulator (RehaMove3; HASOMED, Magdeburg, Germany) set to deliver charge-balanced, symmetric, and biphasic rectangular pulses of 2 ms width (1 ms per phase) [18]. With respect to the abdominal electrodes, the selected stimulating electrode served as anode for the first phase and the cathode for the second phase of the biphasic pulses.

EMG activity was acquired from the right and left L2–L4 innervated quadriceps muscle groups (RQ and LQ) as well as the L5–S2 innervated triceps surae muscle groups (RTS and LTS) [36] by using pairs of surface electrodes placed centrally over the muscle bellies with a distance of 1–2 cm (Figure 1A(ii)). For each lower limb, a wireless two-channel EMG sensor was used to sample the EMG activity of Q and TS at 1 kHz (MuscleLab; Ergotest, Porsgrunn, Norway). Common reference electrodes for both EMG channels of a limb were placed bilaterally on the outer edge of the patella. The skin was cleaned prior to EMG electrode placement to minimize signal noise.

All hardware was controlled by a customized program, developed in Matlab/Simulink (MathWorks, Natick, MA, USA), by using a modified Linux ERT target [37] and a specially created stimulation interface (Python, Kivy). Post-processing analysis was performed in MATLAB (R2021a).

### 2.3. Automatic Determination of Electrodes and Parameters

#### 2.3.1. Automated Stimulation Protocol

Stimulation was applied with the participants lying in the supine position. Starting with the most caudal electrode (position 1), each of the four paraspinal electrodes was selected, one by one, as the stimulating electrode (Figure 1B). From each position, three double stimuli with an inter-pulse-interval of 50 ms were administered with a stimulating amplitude initially set at 5 mA, with 5 s between repetitions. Subsequently, the stimulation amplitude was increased by 5 mA. The same procedure was repeated for stimulation amplitudes up to 75 mA or the individually maximum tolerated amplitude. For each double stimulus, raw EMG activity was recorded from the four muscle groups for time windows of 600 ms starting shortly before the first stimulus (see below).

The automated stimulation protocol could be monitored by the custom user interface, which provided information on the active electrode position at each time as well as a countdown to the application of the next double stimulus. The lower and upper limits of the stimulation amplitude could be manually adjusted to differ from the default values of 5 mA and 75 mA, respectively. The custom user interface additionally provided an online view of the raw EMG data derived from the four muscle groups studied as well as, optionally, the visualization of stimulus-triggered muscle responses and the averaged EMG waveforms derived from the three double stimuli at a given stimulation amplitude (cf. Figure 1C). In addition, a color-coded rating of the EMG responses can be displayed (cf. Section 2.3.3).

#### 2.3.2. EMG Pre-Processing

##### EMG Synchronization

The wirelessly transmitted EMG data, acquired with the two sensors placed on the left and right lower limbs, were first synchronized to the times of double stimulus application. Data from Q and TS of a single limb were acquired by the same sensor and, hence, required no further synchronization. For the synchronization of the EMG data to the double stimuli, stimulation artifacts detected in the raw EMG signals of RQ and LQ were utilized, and time windows from 30 ms before to 300 ms after the first stimulus were extracted. Specifically, the stimulation artifacts were detected by performing non-causal double differentiation of the EMG signals of RQ and LQ by using a discrete approximation of the Laplace’s differential operator Δ:

(1) $$emg_{i,\Delta }\left(n,I,j,\tilde{t}\right)=4\Delta \left(emg_{i,\mathrm{raw}}\left(n,I,j,\tilde{t}\right)\right),\;i\in \left\{\mathrm{LQ},\mathrm{RQ}\right\},\tilde{t}=1,\;\ldots ,600$$

where $emg_{i,\mathrm{raw}}\left(n,k,j,\tilde{t}\right))$ is the raw EMG of the muscle i∈{LQ,RQ} with the corresponding sample index $\tilde{t}$ for the position n∈{1,2,3,4} of the active tSCS electrode and applied stimulation amplitude I=5,10,…≤75mA. The index j∈{1,2,3} describes the repetition number of the double stimuli.

Samples $\tilde{t}$ were marked as potential stimulation artifacts instances ${\tilde{t}}^{*}$, caused by the first stimulus, if they fulfilled the following four conditions.

(2) $$\begin{array}{ccc} \left(C1\right) & & |emg_{i,\Delta }\left(n,I,j,{\tilde{t}}^{*}\right)|>3\mathrm{SD}\left({\left\{emg_{i,\Delta }\left(n,I,j,\tilde{t}\right)\right\}}_{\tilde{t}=300,\cdots ,600}\right), \end{array}$$

(3) $$\begin{array}{ccc} \left(C2\right) & & |emg_{i,\Delta }\left(n,I,j,{\tilde{t}}^{*}+50\right)|>3\mathrm{SD}\left({\left\{emg_{i,\Delta }\left(n,I,j,\tilde{t}\right)\right\}}_{\tilde{t}=300,\cdots ,600}\right), \end{array}$$

(4) $$\begin{array}{ccc} \left(C3\right) & & |emg_{i,\Delta }\left(n,I,j,{\tilde{t}}^{*}+100\right)|\leq 3\mathrm{SD}\left({\left\{emg_{i,\Delta }\left(n,I,j,\tilde{t}\right)\right\}}_{\tilde{t}=300,\cdots ,600}\right), \end{array}$$

(5) (C4)(sign(emgi,raw,mf(n,I,j,t˜∗))≠sign(emgi,raw,mf(n,I,j,t˜∗−1)))∨(C3)(sign(emgi,raw,mf(n,I,j,t˜∗))≠sign(emgi,raw,mf(n,I,j,t˜∗+1))).

Here, the signal $emg_{i,\mathrm{raw},\mathrm{mf}}$ was obtained by subtracting its median from the raw EMG signal in order to remove any present offset levels. Hence, condition (C4) checks for a sign change in the median-free raw EMG signal. The earliest candidate ${\tilde{t}}^{*}$ in the interval $\tilde{t}\in \left[10,200\right]$ was chosen as new time instance (t=0) of the first stimulus and is used to time-align the EMG recordings of the two sensors. Afterwards, the EMG signals were cropped to the new time range −30 to 300 ms for *t*.

If the synchronization failed, i.e., if no time instant could be found at which the conditions C1–C4 were fulfilled, then the corresponding measurement data were discarded, except for the data containing only baseline EMG (noise). Such baseline recordings can occur, e.g., in the case of low stimulation amplitudes (normally <20 mA) and for the most rostral electrode position 4, as stimulation artifacts are barely present. In this case, the first 331 EMG samples were extracted for displaying at the user interface and to capture the noise characteristics. The measurement was declared as baseline EMG when condition (C1) was not fulfilled for any single sample.

##### EMG Pre-Filtering

The median was removed from the cropped and synchronized raw EMG signals for offset correction, and detected stimulation artifacts were blanked (set to 0) for intervals of −2–2 ms and 48–52 ms. A second-order notch filter (Butterworth bandstop filter for the frequency range 43–47 Hz) as well as a first-order low-pass filter (Butterworth filter design with cutoff frequency of 300 Hz) were subsequently non-causally applied to the pre-processed EMG signals. The blanked intervals of the stimulation artifacts were restored in the filtered EMG signals to enable the synchronization and averaging of EMG responses to the three repetitions of double stimuli applied with a given stimulation amplitude and from a given stimulation electrode position.

##### Muscle-Specific Base Noise Level

For further analysis, the noise level ${\bar{emg}}_{i}$ of each muscle *i* at rest was defined by the standard deviation of all EMG samples $emg_{i}\left(n,I,j,t\right)$ for all *n*, *I*, *j*, and 100≤t≤300.

##### Similarity Check and Averaging

For each muscle studied, the filtered EMG signals recorded during the three repetitions of double stimulus application from a given electrode position and with a given stimulation amplitude were checked for similarity by analyzing the root mean square error (RMSE) between the repetitions in the time interval 5–45 ms plus 55–95 ms. Signals were declared as similar if their RMSE was less than 16 times the muscle-specific base noise level ${\bar{emg}}_{i}$. The threshold parameter was determined empirically. Similar EMG signals were then cropped to a time range of −1 to 200 ms relative to the first of the double stimuli of each repetition and averaged to obtain the mean EMG signal $emg_{i,\mathrm{avg}}\left(n,I,t\right)$, with *n* being the electrode position, *I* being the stimulation amplitude, and *t* being the sample index. Non-similar repetitions were discarded. If no similarity was found in at least two repetitions, the respective recording was marked as invalid. Such cases could occur, e.g., when stimulating during unintended movements, or when the initial artifact detection yielded incorrect results. The similarity check prevented such data from falsifying the results of the subsequent automatic evaluation.

#### 2.3.3. Automatic Evaluation of EMG Responses

The averaged EMG signals $emg_{i,\mathrm{avg}}\left(n,I,t\right)$, obtained for a given electrode position *n* and stimulation amplitude *I*, were further analyzed by separately calculating the EMG peak-to-peak amplitudes of the responses to the first and the second of the double stimuli, i.e., $amp_{1,i}\left(n,I\right)$ and $amp_{2,i}\left(n,I\right)$, within time intervals of 10–45 ms following the respective stimuli. The suppression level of the second to the first response was then calculated as follows:

(6) $$sup_{i}\left(n,I\right)=1-amp_{2,i}\left(n,I\right)/amp_{1,i}\left(n,I\right)$$

with i∈{LQ,LTS,RQ,RTS}. The level was limited to the range of [0,1]. A value of one would signify complete suppression, while a value of zero would indicate two responses of same size, i.e., the lack of post-stimulation depression and, hence, the presence of M-waves [29].

The averaged EMG signals of a given muscle were then classified into four cases (cf. Table 2): “no response” if $amp_{1,i}\left(n,I\right)$ was ≤ 50 μV or did not differ from the baseline EMG by at least six standard deviations; “reflex response” if $amp_{1,i}\left(n,I\right)$ was > 50 μV and differed from the baseline EMG by at least six standard deviations with a suppression level $sup_{i}\left(n,I\right)$>60%; “presumed M-wave” if previous conditions on $amp_{1,i}\left(n,I\right)$ were valid but suppression level was ≤60%; and “invalid” if no similar EMG signals were obtained for stimulation from a given electrode position and with given stimulation amplitude. The assigned color codes in Table 2 enable a rating light system in which green indicates the desired reflex responses.

#### 2.3.4. Automatic Delineation of Stimulation Position and Amplitude for tSCS Therapy

Two independent approaches were implemented to identify a suitable electrode position and stimulation amplitude for tSCS therapy, the *ranking approach* considering the color code classification of responses only, and the *cost function approach* additionally considering the obtained EMG peak-to-peak amplitudes and suppression levels.

##### Ranking Approach

Based on the color code classification, a ranking of pairs (*n*—electrode position and *I*—stimulation amplitude) was performed by utilizing a nested search in the following priority (order):

1. At least two green labels present (i.e., reflex responses elicited in at least two of the four muscle groups studied; minimal requirement);
2. Largest number of green labels (i.e., reflex responses);
3. Smallest current level difference between stimulation amplitude *I* and $I^{\prime }$, at which the first green label was obtained (amplitude threshold);
4. Lowest stimulation amplitude *I*.

All electrodes fulfilling the minimal requirement were ranked, with rank “1” indicating best fulfillment of criteria 2–4 within the available search space. For tSCS therapy in spasticity, the best ranked electrode position *n* and a stimulation amplitude corresponding to 90% of the respective stimulation amplitude threshold $I^{\prime }$ [23] were suggested as therapy parameters.

##### Cost Function Approach

In addition to the color code classification, the cost function approach considered the attainable peak-to-peak amplitudes of the EMG responses and the precise suppression levels by using the following cost function.

(7) $$J\left(n,I\right)=\frac{1}{4}\sum_{i\in \{\mathrm{LQ},\mathrm{LTS},\mathrm{RQ},\mathrm{RTS}\}}\frac{amp_{1,i}\left(n,I\right)}{{\bar{amp}}_{1,i}}sup_{i}\left(n,I\right).$$

Here, the response amplitudes of each muscle were normalized with respect to their maximal amplitude ${\bar{amp}}_{1,i}$ found in the corresponding muscle and scaled with the observed suppression ratio.

The electrode position with the largest cost function value *J* was selected for tSCS therapy, favoring electrode positions yielding reflex responses of large peak-to-peak amplitudes. The stimulation amplitude for tSCS for spasticity control was the same as suggested by the ranking approach, i.e., 90% of the amplitude threshold of the selected electrode position.

##### Graphical Visualization

**Figure 2:**
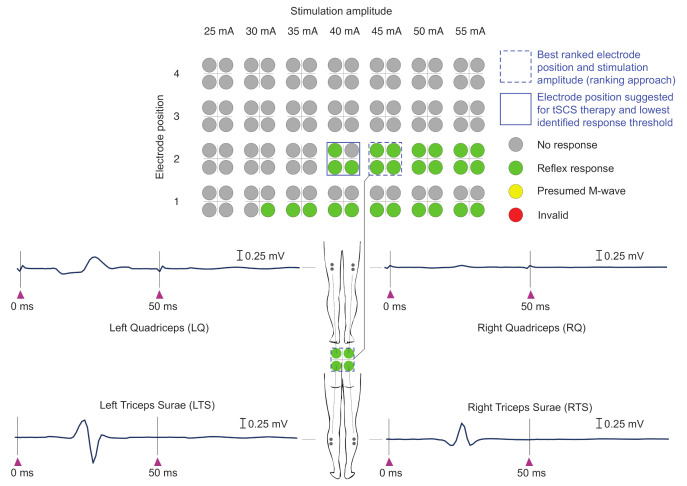
Rating light matrix derived for a neurologically intact individual, C1, along with EMG signals averaged from three repetitions of double stimulus application from electrode position 2 with a stimulation amplitude of 45 mA (best ranked parameter combination). The four color-coded circles represent the results obtained for the four studied muscle groups per electrode position *n* and tested stimulation amplitude *I* (cf. Table 2). In favor of a compact overview, results are only displayed for the stimulation amplitude before the first detected response, in this case from 25 mA instead of from 5 mA, until the highest applied intensity.

**Figure 3:**
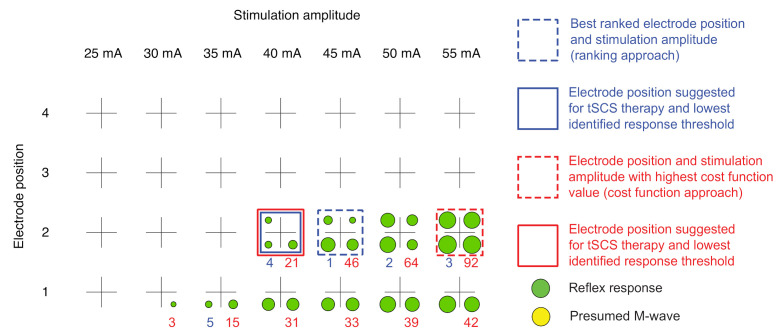
Rating details matrix derived for a neurologically intact individual, C1. In addition to the color codes, reflex peak-to-peak amplitudes are reflected by the size of the colored circles representing the four muscle groups studied. If no response had been elicited, the circle shrank to a dot. Blue numbers are ranks 1–5 as derived from the ranking approach, and red numbers are cost function values *J* calculated in the cost function approach, with higher values signifying favorable results.

The color coded results of the automatic evaluation of the EMG responses $emg_{i,avg}\left(n,I\right)$ are presented as a rating light matrix (Figure 2) for all muscles *i*, electrode positions *n*, and applied stimulation amplitudes *I*. After termination of the stimulation protocol, the electrode position and stimulation amplitude pairs with the highest rank (ranking approach) were marked, and the suggested stimulation amplitude for the tSCS intervention was provided. For a more detailed overview, the rating details matrix was additionally developed, including the color codes as well as details obtained with both the ranking and the cost function approaches (see Figure 3 for an example). The rating detail matrix provided information on the five best ranked electrode positions along with the respective cost function values.

### 2.4. Validation

In order to validate the proposed algorithms for automatic tSCS calibration, all preprocessed averaged EMG signals $emg_{i,\mathrm{avg}}\left(n,I,t\right)$ of our data set were presented to two experts in the field of tSCS (U.S.H. and N.W.). For each participant, the experts annotated the electrode position and stimulation amplitude that they would choose for a sub-threshold tSCS therapy setup (e.g., for treating spasticity). The experts were blinded to the participant’s condition. The annotations were then compared with the results of the automatic determination by the ranking approach and the cost function approach.

## 3. Results

The stimulation and recording setups as well as the algorithms for the calibration of tSCS settings for therapeutic application were successfully and safely applied in all 15 study participants. No adverse events were encountered. Across participants, the complete execution of the stimulation protocol and the determination of stimulation parameters took on average ten min, ranging from 7 to 15 min.

**Figure 4:**
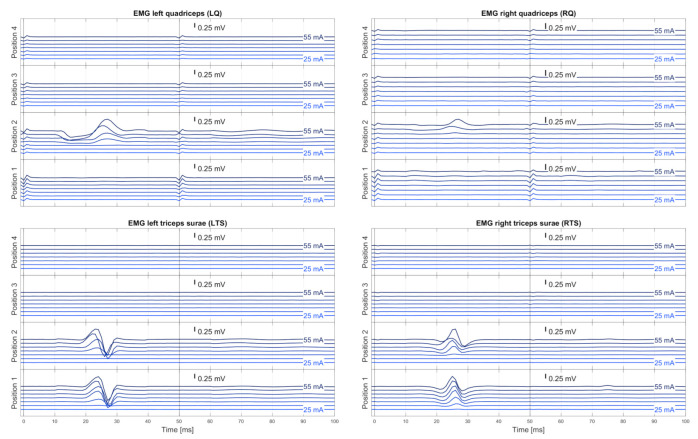
EMG recordings derived from bilateral quadriceps and triceps surae muscle groups during tSCS applied from different rostrocaudal electrode positions with double-stimuli of incremental amplitudes. Exemplary traces derived from a neurologically intact individual, C1, as they were shown to experts. A step-wise increase in stimulation amplitude resulted in the elicitation of responses in bilateral triceps surae by the first pulse of the double stimuli, yet only when applied from the two caudal electrode positions 1 and 2. Bilateral quadriceps responses were present only with stimulation delivered from position 2, hence the best ranked position. Response suppression when the second pulses of the double stimuli were applied after 50 ms identified the responses as reflexes. Each trace averaged from up to three double stimuli per stimulation amplitude; blue-shaded values are stimulation amplitudes.

Exemplary pre-processed averaged EMG signals $emg_{i,\mathrm{avg}}\left(n,I,t\right)$ derived during the automatic execution of the stimulation protocol in one neurologically intact individual and used for expert validation are displayed in Figure 4. The corresponding rating light matrix using color codes for the classification of electrode positions and stimulation amplitudes along with the averaged representation of EMG signals obtained with the best ranked setting (n,I) is shown in Figure 2. In this case, electrode position n=2 was selected since it was the only electrode position resulting in the elicitation of reflex responses in all four studied muscle groups. Furthermore, a stimulation amplitude of 35 mA was suggested for therapy (90% of $I^{\prime }$, amplitude threshold, here 40 mA). The rating details matrix (Figure 3) exhibits that both the ranking approach as well as the cost function approach resulted in the suggestion of the same parameter settings for therapy despite differences in the rankings (stimulation with 45 mA in the case of the ranking approach vs. 55 mA in the case of the cost function approach; see also Table 3).

Across participants, validation revealed a high accuracy of the automatic ranking of stimulation site and amplitude relative to the experts’ classification (Table 3). Specifically, the same electrode position was selected by both experts as well as both evaluation approaches in 11 out of 15 participants. In another two participants in whom no EMG responses were detected even at the maximum applied stimulation amplitudes (participants P3 and P4 with PD), no electrode position was suggested, neither by the experts nor the automatic approaches. In one of the remaining participants (S4), expert #1 selected an electrode that was one position more caudal than the one automatically detected as well as chosen by expert #2. In the other remaining participant (S1), the two experts selected different but neighboring electrode positions, n=2 and n=1, as did the two automatic approaches, also n=2 and n=1. This was the only case in which the ranking approach and the cost function approach did not select the same electrode position.

**Figure 5:**
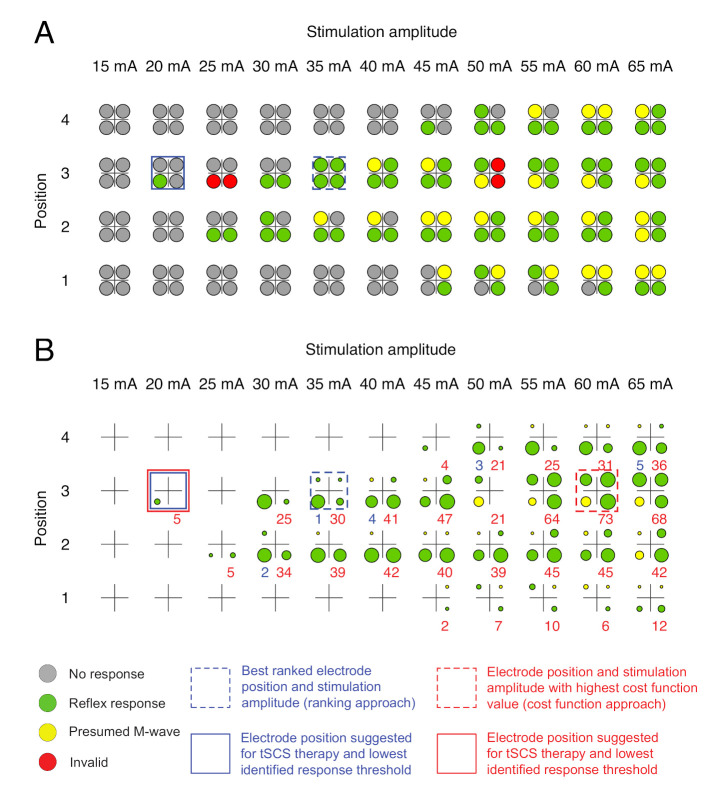
Comparison of ranking based on the two automatic response evaluation approaches applied. (**A**) Rating light matrix and (**B**) rating details matrix derived for participant S2 with SCI. Both approaches resulted in the suggestion of electrode position 3 and identified 20 mA as the lowest response threshold. The four color-coded circles represent the results obtained for the four muscle groups studied (cf. Table 2). In the rating details matrix, the cycle size additionally provides information on the attainable response sizes. If no response had been elicited, the circle shrank to a dot. Blue numbers are ranks 1–5 as derived from the ranking approach, and red numbers are cost function values *J* calculated in the cost function approach, with higher values signifying favorable results.

**Figure A1:**
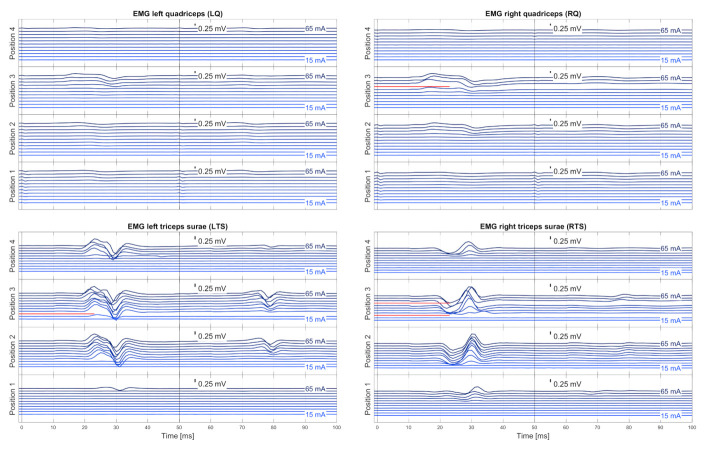
EMG recordings derived from bilateral quadriceps and triceps surae muscle groups during tSCS applied from different rostrocaudal electrode positions with double-stimuli of incremental amplitudes. Exemplary results derived from an individual with SCI, S2. Each trace averaged from up to three double stimuli per stimulation amplitude; blue-shaded values are stimulation amplitudes.

**Figure A2:**
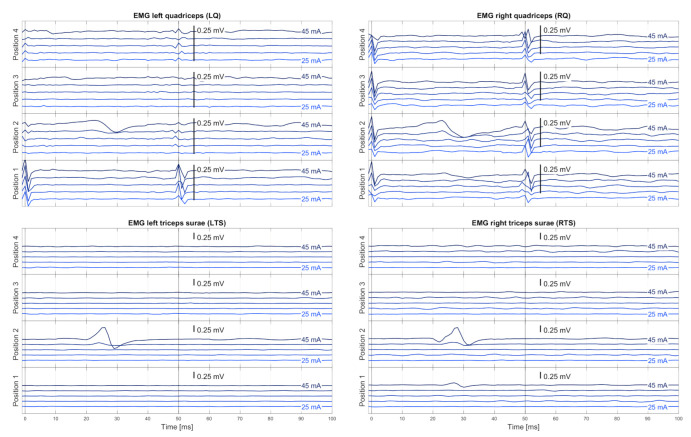
EMG recordings derived from bilateral quadriceps and triceps surae muscle groups during tSCS applied from different rostrocaudal electrode positions with double-stimuli of incremental amplitudes. Exemplary results derived from an individual with PD, P1. Each trace averaged from up to three double stimuli per stimulation amplitude; blue-shaded values are stimulation amplitudes.

**Figure A3:**
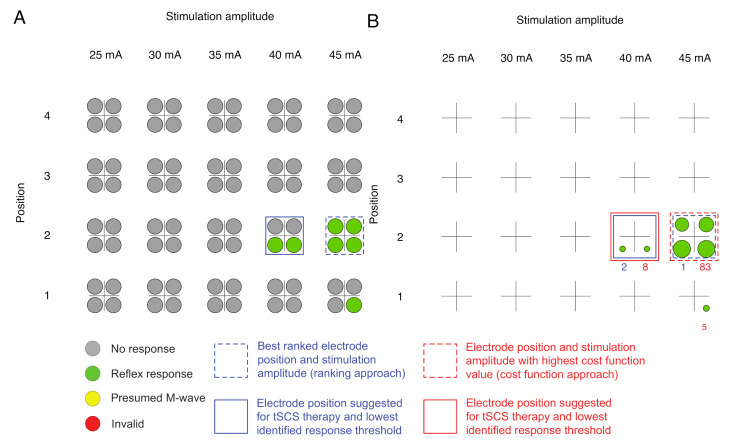
Comparison of ranking based on the two automatic response evaluation approaches applied. (**A**) Rating light matrix and (**B**) rating details matrix derived for participant P1 with PD. Both approaches resulted in the suggestion of electrode position 2 for therapy and identified 40 mA as the lowest response threshold (suggested stimulation amplitude for therapy: 0.9 × 40 mA = 36 mA). The four color-coded circles represent the results obtained for the four muscle groups studied (green, reflex response; cf. Table 2)). In the rating details matrix, the circle size additionally provided information on the attainable response amplitudes. If no response had been elicited, the circle shrank to a dot. Blue numbers are ranks as derived from the ranking approach; red numbers are cost function values *J* calculated in the cost function approach, with higher values signifying favorable results.

With respect to the stimulation amplitude suggested for therapy, congruent values between experts and algorithms were found in six out of the fifteen participants, with another two participants for whom no stimulation amplitudes were suggested (P3 and P4; see above). In participant S1, expert #1 chose the same stimulation amplitude as the cost function approach (25 mA, n=2), and expert #2 agreed with the ranking approach (35 mA, n=1). In four participants (C1, S5, P1, and P5), there was a difference of one increment (i.e., 5 mA) between the four suggested stimulation amplitudes for therapy, and in two participants (S2 and S3), there was a maximum difference of 10 mA. The rating light matrix and the rating details matrix derived for participant S2 are shown in Figure 5, and the corresponding EMG signals are provided in Figure A1 in the Appendix A. Exemplary EMG responses and rating matrices of a PD individual (P1) are shown in Figure A2 and Figure A3. Notably, across participants, only 18 out of 601 evaluated EMG responses were classified as being invalid, corresponding to an error ratio of 3%.

## 4. Discussion

In this article, we presented a novel method that maps expert knowledge on the application of tSCS as a neurorehabilitative method into an automated evaluation algorithm. The developed combined stimulation and evaluation protocol allows for real-time classification of responses evoked by double-stimuli tSCS in several lower limb muscles bilaterally, identifying them either as direct motor responses (M-waves) or as PRM reflexes initiated within afferent fibers of the lumbosacral posterior roots [16,29]. This information is further processed into individual treatment recommendations for electrode position and stimulation amplitude for the application of tSCS in therapeutic settings, e.g., for the control of spinal spasticity [18,20,23,25]. Blinded validations by two independent experts confirmed the accuracy of the automatically derived recommendations. With respect to the electrode position specifically, congruent results were obtained by both evaluation approaches implemented as well as both experts in 13 out of 15 cases (87%).

Our novel approach with an array of four surface electrodes placed longitudinally over the spine to cover mid-lumbar to low-thoracic vertebral levels allowed us to simultaneously evaluate several stimulation sites, rather than having to relocate a single stimulating electrode as in traditional tSCS setups until the required PRM reflex distribution in the lower limb muscles is obtained [18]. This is of specific relevance when treating individuals with severely limited mobility and further helps minimize the time needed to find individually tailored stimulation settings. Other studies of tSCS for neurorehabilitation have used a pragmatic design, placing a single active electrode over vertebral levels T11/T12 in all participants [20,38]. However, previous studies of EES in lower-limb motor control have pointed at the distinctive importance of the specific placement of active electrodes over lumbar and upper sacral spinal cord segments [1,6,27,28]. Consequently, EES parameter settings are thoroughly tested and individually optimized prior to the full implantation of the system for chronic stimulation [14]. Our automated calibration of tSCS may, hence, present a first step towards facilitating individually tailored stimulation setups and maximizing the attainable therapeutic outcomes.

In the present study, the duration for the execution of the automated protocols was approximately 10 min on average. This duration could likely be further reduced by roughly two min by omitting the lower range of stimulation amplitudes (5 mA and 10 mA) at which no responses were evoked in any of our study participants. Furthermore, using a setup with three instead of four rostrocaudally arranged stimulation electrodes, as in participants C5, S5, and P5, would shorten the protocol by another estimated 2 min, resulting in an overall duration of 6 min for the entire procedure. Notably, the most rostral electrode position was not recommended for therapy for any of the individuals tested with four electrodes, neither by the automated calibration nor the two experts. The repeated use of our tSCS setup in an individual patient could additionally limit the search space, i.e., by using less electrodes and test even fewer stimulation amplitudes.

We implemented two evaluation approaches emphasizing different aspects of the detected EMG responses, i.e., the occurrence of PRM reflexes in the majority of studied muscles or the precise amplitude and suppression characteristics, respectively. Both approaches resulted in the same recommendations of electrode positions and the same detection of response thresholds across all but one participant. Both algorithms further provided information on tSCS configurations in addition to the best ranked results that could be alternatively employed should they be perceived as more comfortable by the individual treated. The patient’s perception is a criterion that is not reflected by our automated evaluation approach, but plays a particular role in the tolerance of tSCS therapy, especially in individuals with intact sensory perception in the stimulation area (here, controls and PD patients). By providing the rating light and rating details matrix, we want to enable the user to select other highly ranked tSCS configurations based on individual preferences and criteria, for example, the patient’s perception of continuous stimulation (50 Hz).

Although PD patients often had higher baseline muscle activity than controls and SCI patients, mostly due to dyskinesia as a side effect of dopaminergic medication (e.g., in patient P1), the algorithms were able to robustly detect PRM reflexes and gave reliable recommendations on stimulation parameters in three PD participants. The proposed methods take the baseline muscle activity into account in the used threshold parameters. However, in two individuals with PD, no responses were elicited by tSCS applied from either of the four electrode positions with stimulation amplitudes of up to 65 mA and 75 mA, respectively. A previous study has shown a significant increase in Hoffmann-reflex thresholds in individuals with PD compared to age-matched controls [39], and it can be assumed that similar changes would also affect the PRM reflex. Notably, the individuals with PD in the present study were collectively older than the neurologically intact controls and the individuals with SCI, and studies have shown a lower excitability of the Hoffmann-reflex in elderly adults [40]. These observations suggest that screening tSCS responses, for example, before EES implantation, could add valuable information for the prospective characterization of patients in future clinical trials.

Our algorithms are currently designed to provide recommendations for the application of tSCS at amplitudes below the threshold for the elicitation of PRM reflexes in the lower limb muscles, as utilized in spasticity treatment [23] or for the augmentation of voluntary locomotor activity in incomplete SCI [19]. Future applications could be extended to the recommendation of parameter settings for locomotion therapy or standing and balance training using higher stimulation amplitudes [14,21,26,41,42]. Furthermore, our present approach was optimized for application of tSCS in the supine position. It is known that the body position influences which neural structures are recruited by lumbar tSCS [43]. An adaptation of our method for other body positions such as the upright position in locomotor training seems useful and well possible in the future. Another improvement in the future could be to allow individual adjustments in the selection criteria, which could facilitate more experienced users to set or fine-tune the rules for specific therapy goals (e.g., prioritizing behavior in specific muscles).

The proposed algorithms can be implemented in interpreted higher programming languages such as Python or MATLAB and applied to EMG data from other clinical recording systems. Artifact detection and signal alignment (synchronization) steps can be omitted if no wireless transmission is involved and the stimulator unit provides a trigger output for synchronization with the measurement system. Both features are usually available in present recording and stimulation systems at clinical research centers. In addition, we are currently developing a tablet-controlled setup with an iOS app that uses Bluetooth low energy to communicate wirelessly with EMG sensors and a stimulator for future broader use in rehabilitation centers and physiotherapy practices. This system will directly exploit the algorithms presented in this article.

## 5. Conclusions

In this study, we developed an automated calibration method for tSCS that accurately identified suitable electrode positions and stimulation amplitudes for therapeutic applications. The setup proved to be applicable by non-specialized health professionals, allowing them to individually calibrate tSCS by the use of a comprehensive graphical user interface. Our approach may, hence, provide an easy-to-use and time-effective solution for clinical decision making. These developments may aid the dissemination of tSCS technologies into non-academic environments and broaden the use of tSCS for diagnostic and therapeutic applications.

## Figures and Tables

**Table 1:** Clinical characteristics of study participants.

Group	Subject	Sex	Age	Height	Weight	SCI		PD	
			Years	cm	kg	Level	AIS	H and Y	FD
Control	C1	m	36	184	82	-	-	-	-
(*n* = 5)	C2	f	30	177	65	-	-	-	-
	C3	m	35	190	78	-	-	-	-
	C4	m	33	181	70	-	-	-	-
	C5	f	30	168	56	-	-	-	-
SCI	S1	m	40	182	80	T5-6	A	-	-
(*n* = 5)	S2	m	57	180	74	T5-6	A	-	-
	S3	f	49	179	76	T5-6	B	-	-
	S4	m	58	185	75	C7	B	-	-
	S5	f	31	165	49	T1-3	B	-	-
PD	P1	m	60	180	70	-	-	2	12
(*n* = 5)	P2	f	77	175	68	-	-	2	9
	P3	f	74	172	80	-	-	2	5
	P4	m	70	176	92	-	-	2	14
	P5	m	70	178	72	-	-	2	2

*AIS*, American Spinal Cord Injury Association Impairment Scale; FD, years since first diagnosis; *H and Y*, Hoehn and Yahr disease severity classification [34]; *level*, neurological level of spinal cord injury; *PD*, Parkinson’s disease; *SCI*, spinal cord injury.

**Table 2:** Rating light system for the automatic evaluation of EMG responses.

Color Code		Description	Condition
•	gray	No response	$\left(amp_{1,i}\left(n,I\right)\leq 6\;{\bar{emg}}_{i}\right)\vee \left(amp_{1,i}\left(n,I\right)\leq 50\;\mu V\right)$
•	green	Reflex response	$\left(amp_{1,i}\left(n,I\right)>6\;{\bar{emg}}_{i}\right)\wedge \left(amp_{1,i}\left(n,I\right)>50\;\mu V\right)\wedge sup_{i}\left(n,I\right)>60\%$
•	yellow	Presumed M-wave	$\left(amp_{1,i}\left(n,I\right)>6\;{\bar{emg}}_{i}\right)\wedge \left(amp_{1,i}\left(n,I\right)>50\;\mu V\right)\wedge sup_{i}\left(n,I\right)\leq 60\%$
•	red	Invalid	No similar EMG signals obtained for stimulation from a given electrode position and with given stimulation amplitude

**Table 3:** Comparison of expert’s selection with the results of the automatic tSCS parameter determination.

Subject	Expert’s Selection				Automatic Selection			
	Expert #1		Expert #2		Cost Function		Ranking	
	n	$I^{\prime }$	n	$I^{\prime }$	n	$I^{\prime }$	n	$I^{\prime }$
C1	2	40 mA	2	35 mA	2	40 mA	2	40 mA
C2	2	25 mA	2	25 mA	2	25 mA	2	25 mA
C3	3	25 mA	3	25 mA	3	25 mA	3	25 mA
C4	2	20 mA	2	20 mA	2	20 mA	2	20 mA
C5	3	35 mA	3	35 mA	3	35 mA	3	35 mA
S1	2	25 mA	1	35 mA	2	25 mA	1	35 mA
S2	3	30 mA	3	30 mA	3	20 mA	3	20 mA
S3	2	45 mA	2	40 mA	2	35 mA	2	35 mA
S4	2	25 mA	3	25 mA	3	25 mA	3	25 mA
S5	2	25 mA	2	20 mA	2	20 mA	2	20 mA
P1	2	45 mA	2	40 mA	2	40 mA	2	40 mA
P2	1	45 mA	1	45 mA	1	45 mA	1	45 mA
P3	-	-	-	-	-	-	-	-
P4	-	-	-	-	-	-	-	-
P5	1	35 mA	1	35 mA	1	40 mA	1	40 mA

*C*, neurologically intact control; *I’*, stimulation amplitude threshold; *n*, number of electrode position (cf. Figure 1A); *P*, individual with Parkinson’s disease; *S*, individual with SCI; -, no response elicited with stimulation amplitudes up to 75 mA; hence, no electrode and stimulation amplitude were suggested. The stimulation amplitude for tSCS therapy is then set to 90% of the identified thresholds *I’*.

## Data Availability

The data presented in this study are available upon reasonable request from the corresponding author.

## Abbreviations

The following abbreviations are used in this manuscript:

AIS	American Spinal Cord Injury Association Impairment Scale;
EES	Epidural electrical stimulation;
EMG	Electromyography;
FD	Years since first diagnosis of PD;
H and Y	Hoehn and Yahr scale;
LQ	Left quadriceps;
LTS	Left tricpes surae;
PD	Parkinson’s disease;
PRM reflex	Posterior root-muscle reflex;
RQ	Right quadriceps;
RTS	Right tricpes surae;
SCI	Spinal cord injury;
tSCS	Transcutaneous spinal cord stimulation.

## References

[1] Wagner F.B., Mignardot J.B., Le Goff-Mignardot C.G., Demesmaeker R., Komi S., Capogrosso M., Rowald A., Seáñez I., Caban M., Pirondini E. (2018). Targeted neurotechnology restores walking in humans with spinal cord injury. Nature.

[2] Angeli C.A., Boakye M., Morton R.A., Vogt J., Benton K., Chen Y., Ferreira C.K., Harkema S.J. (2018). Recovery of over-ground walking after chronic motor complete spinal cord injury. N. Engl. J. Med..

[3] Gill M.L., Grahn P.J., Calvert J.S., Linde M.B., Lavrov I.A., Strommen J.A., Beck L.A., Sayenko D.G., Van Straaten M.G., Drubach D.I. (2018). Neuromodulation of lumbosacral spinal networks enables independent stepping after complete paraplegia. Nat. Med..

[4] Minassian K., Jilge B., Rattay F., Pinter M., Binder H., Gerstenbrand F., Dimitrijevic M.R. (2004). Stepping-like movements in humans with complete spinal cord injury induced by epidural stimulation of the lumbar cord: Electromyographic study of compound muscle action potentials. Spinal Cord..

[5] Ladenbauer J., Minassian K., Hofstoetter U., Dimitrijevic M.R., Rattay F. (2010). Stimulation of the human lumbar spinal cord with implanted and surface electrodes: A computer simulation study. IEEE Trans. Neural Syst. Rehabil. Eng..

[6] Pinter M., Gerstenbrand F., Dimitrijevic M. (2000). Epidural electrical stimulation of posterior structures of the human lumbosacral cord: 3. Control of spasticity. Spinal Cord..

[7] Angeli C., Edgerton V., Gerasimenko Y., Harkema S. (2014). Altering spinal cord excitability enables voluntary movements after chronic complete paralysis in humans. Brain.

[8] Minassian K., McKay W., Binder H., Hofstoetter U. (2016). Targeting Lumbar Spinal Neural Circuitry by Epidural Stimulation to Restore Motor Function After Spinal Cord Injury. Neurotherapeutics.

[9] de Andrade E.M., Ghilardi M.G., Cury R.G., Barbosa E.R., Fuentes R., Teixeira M.J., Fonoff E.T. (2015). Spinal cord stimulation for Parkinson’s disease: A systematic review. Neurosurg. Rev..

[10] Pinto de Souza C., Hamani C., Oliveira Souza C., Lopez Contreras W.O., dos Santos Ghilardi M.G., Cury R.G., Reis Barbosa E., Jacobsen Teixeira M., Talamoni Fonoff E. (2017). Spinal cord stimulation improves gait in patients with Parkinson’s disease previously treated with deep brain stimulation. Mov. Disord..

[11] de Lima-Pardini A., Coelho D., Pinto de Souza C., Souza C.O., dos Santos Ghilardi M.G., Garcia T., Voos M., Milosevic M., Hamani C., Teixeira L.A. (2018). Effects of spinal cord stimulation on postural control in Parkinson’s disease patients with freezing of gait. Elife.

[12] Fonoff E., de Lima-Pardini A., Coelho D., Monaco B., Machado B., Pinto de Souza C., dos Santos Ghilardi M., Hamani C. (2019). Spinal cord stimulation for freezing of gait: From bench to bedside. Front. Neurol..

[13] Prasad S., Aguirre-Padilla D., Poon Y., Kalsi-Ryan S., Lozano A.M., Fasano A. (2020). Spinal Cord Stimulation for Very Advanced Parkinson’s Disease: A 1-Year Prospective Trial. Mov. Disord..

[14] Hofstoetter U.S., Freundl B., Binder H., Minassian K. (2018). Spinal cord stimulation as a neuromodulatory intervention for altered motor control following spinal cord injury. Advanced Technologies for the Rehabilitation of Gait and Balance Disorders.

[15] Courtine G., Harkema S., Dy C., Gerasimenko Y.P., Dyhre-Poulsen P. (2007). Modulation of multisegmental monosynaptic responses in a variety of leg muscles during walking and running in humans. J. Physiol..

[16] Minassian K., Hofstoetter U., Frank R., Dimitrijevic M., Kakulas B., McKay W., Vrbová G. (2011). Transcutaneous lumbar posterior root stimulation for motor control studies and modification of motor activity after spinal cord injury. Restorative Neurology of Spinal Cord Injury.

[17] Hofstoetter U., Freundl B., Binder H., Minassian K. (2018). Common neural structures activated by epidural and transcutaneous lumbar spinal cord stimulation: Elicitation of posterior root-muscle reflexes. PLoS ONE.

[18] Hofstoetter U., McKay W., Tansey K., Mayr W., Kern H., Minassian K. (2014). Modification of spasticity by transcutaneous spinal cord stimulation in individuals with incomplete spinal cord injury. J. Spinal Cord. Med..

[19] Hofstoetter U., Krenn M., Danner S., Hofer C., Kern H., McKay W.B., Mayr W., Minassian K. (2015). Augmentation of Voluntary Locomotor Activity by Transcutaneous Spinal Cord Stimulation in Motor-Incomplete Spinal Cord-Injured Individuals. Artif. Organs.

[20] Estes S.P., Iddings J.A., Field-Fote E.C. (2017). Priming neural circuits to modulate spinal reflex excitability. Front. Neurol..

[21] Gad P., Gerasimenko Y., Zdunowski S., Turner A., Sayenko D., Lu D., Edgerton V. (2017). Weight Bearing Over-ground Stepping in an Exoskeleton with Non-invasive Spinal Cord Neuromodulation after Motor Complete Paraplegia. Front. Neurosci..

[22] Sayenko D.G., Rath M., Ferguson A.R., Burdick J.W., Havton L.A., Edgerton V.R., Gerasimenko Y.P. (2019). Self-Assisted Standing Enabled by Non-Invasive Spinal Stimulation after Spinal Cord Injury. J. Neurotrauma.

[23] Hofstoetter U.S., Freundl B., Danner S.M., Krenn M.J., Mayr W., Binder H., Minassian K. (2020). Transcutaneous Spinal Cord Stimulation Induces Temporary Attenuation of Spasticity in Individuals with Spinal Cord Injury. J. Neurotrauma.

[24] Meyer C., Hofstoetter U.S., Hubli M., Hassani R.H., Rinaldo C., Curt A., Bolliger M. (2020). Immediate Effects of Transcutaneous Spinal Cord Stimulation on Motor Function in Chronic, Sensorimotor Incomplete Spinal Cord Injury. J. Clin. Med..

[25] Hofstoetter U., Freundl B., Lackner P., Binder H. (2021). Transcutaneous Spinal Cord Stimulation Enhances Walking Performance and Reduces Spasticity in Individuals with Multiple Sclerosis. Brain Sci..

[26] Roberts B., Atkinson D., Manson G., Markley R., Kaldis T., Britz G., Horner P., Vette A., Sayenko D. (2021). Transcutaneous spinal cord stimulation improves postural stability in individuals with multiple sclerosis. Mult. Scler. Relat. Disord..

[27] Hofstoetter U.S., Perret I., Bayart A., Lackner P., Binder H., Freundl B., Minassian K. (2021). Spinal motor mapping by epidural stimulation of lumbosacral posterior roots in humans. iScience.

[28] Murg M., Binder H., Dimitrijevic M. (2000). Epidural electric stimulation of posterior structures of the human lumbar spinal cord: 1. muscle twitches—A functional method to define the site of stimulation. Spinal Cord..

[29] Minassian K., Persy I., Rattay F., Dimitrijevic M.R., Hofer C., Kern H. (2007). Posterior root-muscle reflexes elicited by transcutaneous stimulation of the human lumbosacral cord. Muscle Nerv..

[30] Hofstoetter U.S., Minassian K., Hofer C., Mayr W., Rattay F., Dimitrijevic M.R. (2008). Modification of reflex responses to lumbar posterior root stimulation by motor tasks in healthy subjects. Artif. Organs.

[31] Calvert J.S., Grahn P.J., Strommen J.A., Lavrov I.A., Beck L.A., Gill M.L., Linde M.B., Brown D.A., Van Straaten M.G., Veith D.D. (2019). Electrophysiological Guidance of Epidural Electrode Array Implantation over the Human Lumbosacral Spinal Cord to Enable Motor Function after Chronic Paralysis. J. Neurotrauma.

[32] Rattay F., Minassian K., Dimitrijevic M. (2000). Epidural electrical stimulation of posterior structures of the human lumbosacral cord: 2. quantitative analysis by computer modeling. Spinal Cord..

[33] Capogrosso M., Wenger N., Raspopovic S., Musienko P., Beauparlant J., Luciani L.B., Courtine G., Micera S. (2013). A computational model for epidural electrical stimulation of spinal sensorimotor circuits. J. Neurosci..

[34] Hoehn M.M., Yahr D. (1967). Parkinsonism: Onset, progression, and mortality. Neurology.

[35] Salchow-Hömmen C., Dikau C., Mueller P., Hofstoetter U., Kühn A., Schauer T., Wenger N. Characterization of optimal electrode configurations for transcutaneous spinal cord stimulation. Proceedings of the IFESS Conference at Rehab Week.

[36] Sayenko D., Atkinson D., Dy C., Gurley K., Smith V., Angeli C., Harkema S., Edgerton V., Gerasimenko Y. (2015). Spinal segment-specific transcutaneous stimulation differentially shapes activation pattern among motor pools in humans. J. Appl. Physiol..

[37] Sojka M., Píša P. Usable Simulink Embedded Coder Target for Linux. Proceedings of the 16th Real Time Linux Workshop.

[38] Estes S., Zarkou A., Hope J.M., Suri C., Field-Fote E.C. (2021). Combined Transcutaneous Spinal Stimulation and Locomotor Training to Improve Walking Function and Reduce Spasticity in Subacute Spinal Cord Injury: A Randomized Study of Clinical Feasibility and Efficacy. J. Clin. Med..

[39] Pierantozzi M., Palmieri M., Galati S., Stanzione P., Peppe A., Tropepi D., Brusa L., Pisani A., Moschella V., Marciani M.G. (2008). Pedunculopontine nucleus deep brain stimulation changes spinal cord excitability in Parkinson’s disease patients. J. Neural. Transm..

[40] Baudry S., Penzer F., Duchateau J. (2014). Input-output characteristics of soleus homonymous Ia afferents and corticospinal pathways during upright standing differ between young and elderly adults. Acta Physiol..

[41] Minassian K., Hofstoetter U.S., Danner S.M., Mayr W., Bruce J.A., McKay W.B., Tansey K.E. (2016). Spinal rhythm generation by step-induced feedback and transcutaneous posterior root stimulation in complete spinal cord–injured individuals. Neurorehabil. Neural. Repair..

[42] McHugh L., Miller A., Leech K., Salorio C., Martin R. (2020). Feasibility and utility of transcutaneous spinal cord stimulation combined with walking-based therapy for people with motor incomplete spinal cord injury. Spinal Cord. Ser. Cases.

[43] Danner S., Krenn M., Hofstoetter U., Toth A., Mayr W., Minassian K. (2016). Body position influences which neural structures are recruited by lumbar transcutaneous spinal cord stimulation. PLoS ONE.

